# Supplementary material for: Monitoring adherence to pharmacological therapy and follow-up examinations among patients with type 2 diabetes in community pharmacies. Results from an experience in Italy
Source: PLoS One. 2021 Sep 7;16(9):e0256478. doi: 10.1371/journal.pone.0256478 (PMC8423241; doi:10.1371/journal.pone.0256478)
Supplement: S1 File — (DOC) [file pone.0256478.s001.doc]

# Appendix 1. Patient questionnaires

| **Socio-demographic characteristics** | | | |
| --- | --- | --- | --- |
| Gender | | M | F |
| Age (in completed years) | | ----- | |
| How many people does your family consist of (excluding yourself)? | | ----- | |
|  |  | YES | NO |
| In case of need, can your family count on the personal help of other non co-habiting people? | Relatives |  |  |
|  | Friends |  |  |
|  | Neighbours |  |  |
|  | Others on a voluntary basis |  |  |
|  | Others for a fee |  |  |
| What is your highest educational qualification? | None |  |  |
|  | Primary school diploma |  |  |
|  | Middle school diploma |  |  |
|  | 2-3 yrs. High school diploma |  |  |
|  | 4-5 yrs. High school diploma |  |  |
|  | University degree or more |  |  |

| **Adherence to guidelines for clinical follow-up examinations** | | | |
| --- | --- | --- | --- |
| How long have you been diagnosed with diabetes? | Years  ----- | | |
| **In the past 6 months:** | Yes | No | Don’t know |
| Have you measured your blood pressure? |  |  |  |
| Have you measured your glycated haemoglobin? |  |  |  |
| **In the past year:** |  |  |  |
| Were you seen by your general practitioner? |  |  |  |
| Were you seen by a diabetologist? |  |  |  |
| Did a doctor or a nurse perform a foot examination? |  |  |  |
| Have you measured your blood cholesterol level? |  |  |  |
| Have you measured your urine albumin level? |  |  |  |
| **In the past 2 years:** |  |  |  |
| Were you seen by an ophthalmologist or did you undergo an observation of the fundus oculi? |  |  |  |

| **Adherence to pharmacological therapies** | | |
| --- | --- | --- |
| How long have you been on pharmacological therapy for diabetes? | Months  ----- | Years  ----- |
| Have you had to go to the Emergency Room or hospital for diabetes-related problems in the past year?  If yes, how many times? ER_____ H________ | YES | NO |
| Do you take medicines for other chronic diseases?  If yes, what disease? |  |  |
| **(one sheet for each class of medications)** |  |  |
| **In the past month:** | YES | NO |
| Have your doctor changed your prescription? |  |  |
| Do you ever forget to take your medicine? |  |  |
| Have you ever voluntary stopped taking your medicine? |  |  |
| Have you ever voluntary changed your prescription because you felt worse? |  |  |
| Have you ever voluntary changed your prescription because you felt better? |  |  |

#

# 
