# Supplementary material for: Monitoring adherence to pharmacological therapy and follow-up examinations among patients with type 2 diabetes in community pharmacies. Results from an experience in Italy
Source: PLoS One. 2021 Sep 7;16(9):e0256478. doi: 10.1371/journal.pone.0256478 (PMC8423241; doi:10.1371/journal.pone.0256478)
Supplement: S2 File — (DOCX) [file pone.0256478.s002.docx]

**Appendix 2. Pharmacists satisfaction questionnaire**

- Indicate 3 positive aspects regarding this project:

1………………….

2………………….

3………………….

- Indicate 3 negative aspects regarding this project:

1………………….

2………………….

3………………….

- Do you have any suggestions for improving the process? _____________________________________________________________________
