# Supplementary figures and images for: Monitoring adherence to pharmacological therapy and follow-up examinations among patients with type 2 diabetes in community pharmacies. Results from an experience in Italy
Source: PLoS One. 2021 Sep 7;16(9):e0256478. doi: 10.1371/journal.pone.0256478 (PMC8423241; doi:10.1371/journal.pone.0256478)

## S3 File. Study flow chart

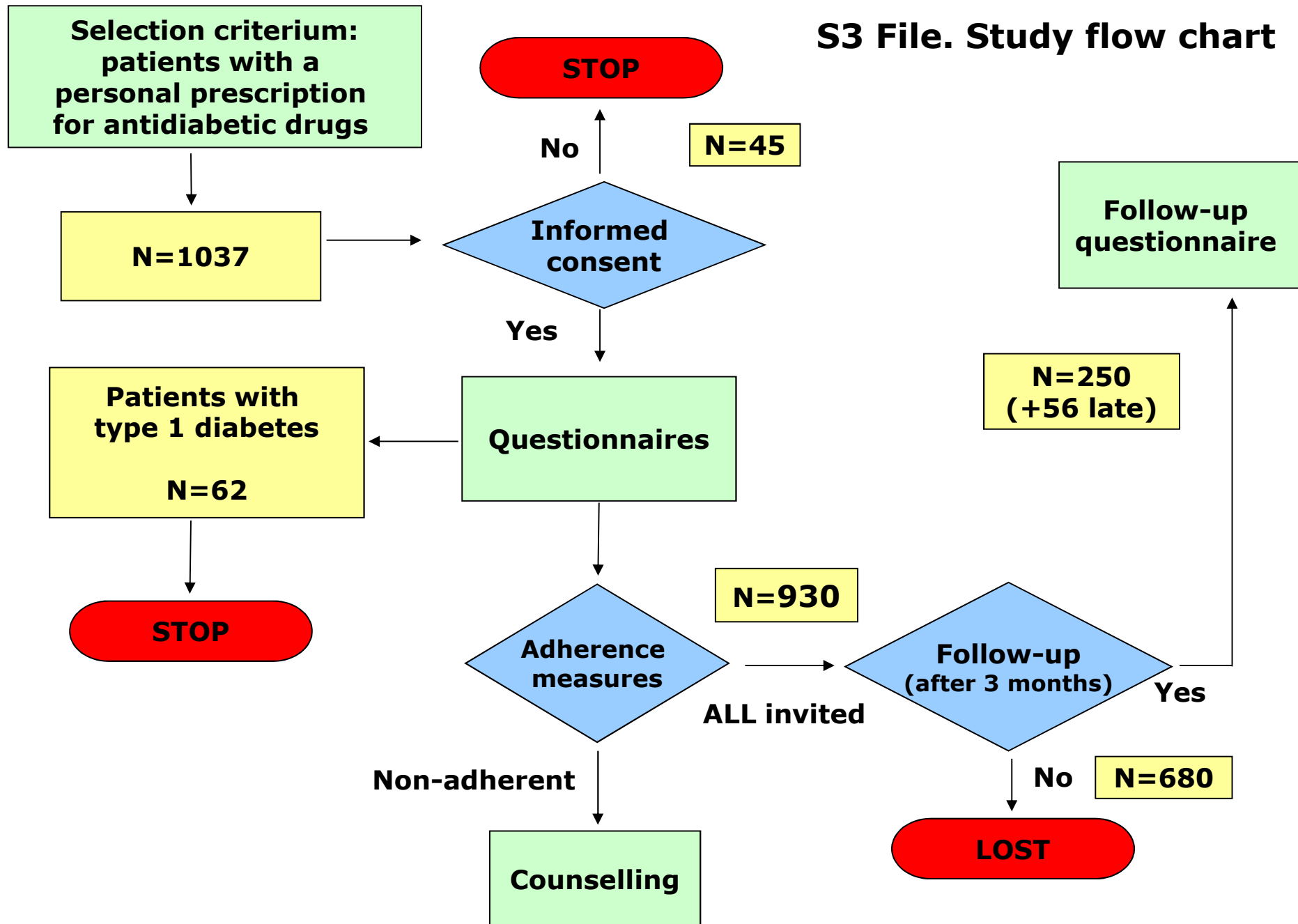

Supplement: S3 File — (PDF) [file pone.0256478.s003.pdf]
