# Supplementary material for: Monitoring adherence to pharmacological therapy and follow-up examinations among patients with type 2 diabetes in community pharmacies. Results from an experience in Italy
Source: PLoS One. 2021 Sep 7;16(9):e0256478. doi: 10.1371/journal.pone.0256478 (PMC8423241; doi:10.1371/journal.pone.0256478)
Supplement: Attachment — Submitted filename: PLOS ONE.docx [file pone.0256478.s005.docx]

Dear Authors,

Manuscript, named as “Monitoring adherence to clinical guidelines among patients with type 2 diabetes in community pharmacies. Results from an experience in Italy” was sended me to review.

Study is very valuable in terms of study design and contribution to the literature.
Some of my suggestions are as follows;

-In introduction, a general information can be given as a result of the literature review on the subject.

-There are some grammatical and writing mistakes, please correct them. Apply to an English editing center.

- Have you used a method for sample selection? Please explain.

- When separating decimals, use dots instead of commas (in table 1).

-Include the superior aspects and differences of your study at the end of the article's discussion section.

-You can expand the discussion according to results.

-Please add a ”conclusion” subheading at the end of the “discussion”.

- There are references that are not in the journal writing format. (Check out the Author Guidelines)
